# Practice Considerations for Adapting in-Person Groups to Telerehabilitation

**DOI:** 10.5195/ijt.2021.6374

**Published:** 2021-06-22

**Authors:** Allison M. Gustavson, Michelle R. Rauzi, Molly J. Lahn, Hillari S.N. Olson, Melissa Ludescher, Stephanie Bazal, Elizabeth Roddy, Christine Interrante, Estee Berg, Jennifer P. Wisdom, Howard A. Fink

**Affiliations:** 1 Veterans Affairs Health Services Research and Development Center for Care Delivery and Outcomes Research, Veterans Affairs Healthcare System, Minneapolis, MN, USA; 2 Physical Therapy Program, Department of Physical Medicine and Rehabilitation, University of Colorado, Aurora, CO, USA; 3 Physical Medicine and Rehabilitation, Minneapolis Veterans Affairs Health Care System, Minneapolis, MN 5, USA; 4 Wisdom Consulting, New York, New York, NY, USA; 5 Geriatric Research Education and Clinical Center, Veterans Affairs Healthcare System, Minneapolis, MN, USA; 6 Department of Medicine, University of Minnesota, Minneapolis, MN, USA

**Keywords:** Clinical practice, Group, Implementation, Multi-participant, Telehealth, Telerehabilitation

## Abstract

The Coronavirus-2019 (COVID-19) pandemic has shifted research and healthcare system priorities, stimulating literature on implementation and evaluation of telerehabilitation for a variety of patient populations. While there is substantial literature on individual telerehabilitation, evidence about group telerehabilitation remains limited despite its increasing use by rehabilitation providers. Therefore, the purpose of this manuscript is to describe our expert team's consensus on practice considerations for adapting in-person group rehabilitation to group telerehabilitation to provide rapid guidance during a pandemic and create a foundation for sustainability of group telerehabilitation beyond the pandemic's end.

The risk for both long-term disability and hospitalization can be significantly attenuated with timely rehabilitation (Falvey et al., 2019; Gill et al., 2018; Guralnik et al., 1995; Hoyer et al., 2013, 2014). The Coronavirus-2019 (COVID-19) pandemic has reduced access to rehabilitation because of outpatient clinic closures, delay or deferral of home services, and patients’ reluctance to be admitted to nursing homes for short-term rehabilitation for fear of contracting COVID-19 (Falvey et al., 2020; Gustavson et al., 2020, 2021). As such, the Veterans Healthcare Administration (VHA) has expanded telerehabilitation nationally to decrease time to post-hospitalization rehabilitation, reach rural areas with few providers, and minimize viral spread by reducing in-person contact.

Telerehabilitation broadly describes the use of electronic information, technology, and different communication mediums (e.g., phone, video, text messaging, email, wearable technologies) to support the delivery of rehabilitation services that serve a single individual or multiple patients (i.e., group) (Brennan et al., 2010; Richmond et al., 2017). Specifically, group telerehabilitation enables providers to reach a greater number of patients and leverage social connectivity to empower patients to achieve functional goals (Banbury et al., 2018; Yalom & Leszcz, 2020). Group telerehabilitation occurs in varying formats including provider connection with a group of patients at one physical location (e.g., community-based outpatient clinic) or, as described in this paper, patients are each located in different physical locations. Physical and occupational therapists employing group telerehabilitation may deliver interventions including patient education (e.g., fall risk reduction strategies, pain management), strengthening exercises, functional activities (e.g., transfer training, stair navigation), and balance training.

The COVID-19 pandemic has accelerated the previously slow adoption of telerehabilitation. As a result, many providers, clinics, and healthcare systems are rapidly readjusting and providing new modes of care delivery with little evidence on what types of care delivery are effective and how to deliver this care (i.e., practice considerations). A small but growing body of literature suggests group telerehabilitation has promising outcomes across rehabilitation professions including within physical therapy and speech therapy (Cox et al., 2018; Hwang et al., 2017; Jennings et al., 2020; Kyrdalen et al., 2014; Lin et al., 2014; Pitt et al., 2019; VanRavenstein & Davis, 2018; Wong et al., 2019). Yet uncertainty exists in how telerehabilitation groups differ from in-person groups, including how to adapt them to ensure efficient delivery and quality care. Lack of clear practice considerations increases the risk for unwarranted practice variation that potentially contributes to poor patient and system-level outcomes (Atsma et al., 2020). Establishing practice considerations is an important step in ensuring quality and consistency of patient care by supplying providers with guidance when uncertainty exists (Woolf et al., 1999).

Therefore, the purpose of this paper is to describe our expert team's process to reach a consensus on practice considerations for adapting in-person group to group telerehabilitation. The practice considerations outlined in this paper are framed from a physical therapy perspective, though the considerations may be transferrable to other rehabilitation providers. Although the development of these practice considerations was predicated by necessity within VHA, we anticipate they will translate across healthcare systems and help providers increase consistency and quality of rehabilitation services delivered to a variety of patient populations.

## METHODS

We formed an expert team to meet and iteratively develop practice considerations for adapting in-person rehabilitation groups to telerehabilitation groups. The meeting agendas were guided by the Model for Adaptation Design and Impact (MADI) framework (Kirk et al., 2020). The MADI framework describes adaptation and guides decision-making around adaptations to better understand their impact on implementation strategies and outcomes. The MADI framework identifies what was adapted, the nature of the adaptation, for whom the adaptation was made, reasons for the adaptation, and when the adaptation occurred (Kirk et al., 2020).

### EXPERT TEAM

Our expert team consisted of providers at the Minneapolis Veterans Affairs Health Care System and the Denver Rocky Mountain Regional Veterans Affairs Medical Center. We invited all physical therapy providers at the Minneapolis Veterans Affairs Health Care System to participate, and interested individuals submitted a statement of interest to physical therapy leadership for consideration. From these interest statements, leadership granted eight physical therapists protected time for participation in workgroup meetings. The group also sought the advice of a physical therapist from the Denver Rocky Mountain Regional Veterans Affairs Medical Center through the individual's involvement in a complementary, funded project on group telerehabilitation. All nine physical therapists possess doctorates in physical therapy (DPT), three are board-certified specialists in either geriatrics or neurology, one is a Doctor of Philosophy (Rehabilitation Science) trainee, and two have additional PhDs (Rehabilitation Science, Mind-Body Medicine). The clinical experience of the physical therapists ranges from 2–23 years and covers multiple settings including acute care, post-acute care, outpatient, pulmonary rehabilitation, and cardiac rehabilitation. One member of the expert team is a research psychologist and expert consultant in interdisciplinary project management. The final team member is an internal medicine physician and clinical researcher.

Six physical therapy team members have direct experience conducting in-person and/or telerehabilitation groups. These members have experience conducting in-person group rehabilitation sessions that focus on Lee Silverman Voice Treatment (LVST) BIG® for patients with Parkinson's disease, balance for those at risk for falls, adapted chair and mat-based yoga, Tai Chi, and return to activity following an episode of back pain. Our telerehabilitation practice experience consists of delivering one-on-one physical therapy evaluation and treatment to Veterans with a variety of neuromuscular and musculoskeletal impairments. Our telerehabilitation group-based experience includes delivery of physical therapy interventions (e.g., high-intensity interval training for patients with Parkinson's disease and parkinsonism, balance training, aerobic conditioning, adapted chair and mat yoga, Tai Chi, and coaching to improve physical activity) to older Veterans (≥ 50 years of age) with multimorbidity (≥3 medical comorbidities), and physical function impairments. These Veterans may have a progressive neurological disorder, chronic pain, mental health conditions, cardiovascular and/or pulmonary diagnosis, endocrine disorders (e.g., diabetes), and/or a subacute or chronic musculoskeletal condition (e.g., osteoarthritis, chronic back pain).

### ADAPTATION OF PRACTICE CONSIDERATIONS FOR IN-PERSON TO TELEREHABILITATION GROUPS

The team met virtually six times for one hour each over a 3-month period (November 2020–January 2021). The first author led meetings with a structured agenda that followed the components of program development and then program implementation over a chronological episode of care. The meeting lead used semi-structured questions guided by the MADI framework (Kirk et al., 2020), including targeted probing questions to identify what rehabilitation providers should know, what they should do, and what resources they might need for a corresponding adaptation. Each meeting began with a summary of the previous meeting's discussion and an opportunity to raise additional comments or concerns on the developing practice considerations until we reached a consensus. We determined a consensus was achieved when no further objections about practice considerations were raised. Following the development of broad practice considerations, we discussed the specifics needed to make implementation possible in the context of the VHA. This manuscript presents the general practice considerations developed that can be translated to the context of other healthcare systems.

## RESULTS

The expert team formed a consensus on the key components and practice considerations for adapting an in-person rehabilitation group to a telerehabilitation group. We mapped adaptations to the MADI framework (Kirk et al., 2020) and classified them under program development or program implementation (Table 1). For each component of program development adaptations and program implementation adaptations, we describe the in-person group rehabilitation process, recommendations for adaptations, and an explanation of reasons for adaptations.

**Table 1:** Identified Adaptations for Development and Implementation of an In-person Group to Telerehabilitation Group

	In-Person Group Processes	Recommended Adaptations for Group Telerehabilitation	Reason for Adaptation for Group Telerehabilitation
**Program Development**			
Identify Eligibility Criteria	Ensure patients can perform activities with minimal supervision.	Consider non-clinical factors including access to needed technology and patient or caregiver capability of managing it, and environmental factors.	Patients need the ability to manage steps to connect to virtual sessions and access to a device, internet connection, and a safe physical space to perform unsupervised rehabilitation activities.
Establish Emergency Protocols	Follow intra-facility emergency policies and procedures.	1) Verify physical location. 2) Verify emergency contact. 3) Ascertain if additional individuals are physically present. 4) Identify the dispatch number.	Provider may need to call for emergency assistance on behalf of the patient.
Identify Outcome Measures	Evaluate measure constructs and psychometric properties to finalize outcome measures.	Evaluate measures that can be assessed virtually.	Many outcome measures are not validated for virtual administration, requiring careful consideration of advantages, disadvantages, and reproducibility of different outcomes.
Identify Maximum Group Size	8–10 patients (5–10:1 patient: provider ratio)	6–8 patients (max of 4:1 ratio of patients to providers)	For novel group curricula, fewer patients overall and/or a lower ratio of patients: providers is desired. For established telerehabilitation groups with familiar curricula, higher patient to provider ratios and a higher maximum number of patients may be possible.
Provide Staff Training	Evidence and resources for reliable administration of tests/measures are based on in-person administration. Current processes for conducting groups are not limited by technology and communication issues.	Provide telerehabilitation-specific training on virtual selection, administration, and interpretation of tests and measures and best practices regarding the delivery of virtual group interventions. Create training for assistants.	Administration of familiar tests/measures within a virtual format is new to providers. An additional assistant was trained to address pre-session orientation and check-in including safety verification, as well as to assist with in-session technical issues that may arise.
**Program Implementation**			
Measure Patient Response to Program	Assess outcome measures in-person.	Assess outcome measures virtually.	Anticipate a virtual evaluation or discharge and, as such, rely more on patient-report surveys and objective measures that are reproducible in the virtual setting (e.g., using 30-second sit to stand to assess gross lower extremity strength rather than using manual muscle testing).
Conduct Pre-Program Orientation	Conduct orientation as a group and include a tour and outline of group expectations.	Conduct orientation individually and include expectations for conduct during the virtual group, use of signals during the session, troubleshooting technical issues, and use of tech support.	Patients have large variation in technological experience and capabilities. Patients need to be aware of additional expectations for conduct in a virtual setting to ensure safety and privacy for all members of the group.
Prepare for the Session	Sessions are not limited by technical issues	Provide written communication in advance of the first session (e.g., technology access/troubleshooting, equipment needs). Open the virtual room early and start check-in with patients.	Build in extra time for technical issues and safety verification.
Conduct the Session	Providers can individually observe patients and offer hands-on cueing.	Providers participate in the activities (e.g., modeling), simplify and repeat tasks, and verbalize cues to modify interventions to account for the minimal visibility of the patient on the screen and inability to provide hands-on cueing. Two personnel were present (provider and an assistant) with clear roles/responsibilities outlined.	Accommodates different levels of functional abilities. An assistant addresses non-clinical questions, monitors the chat (if applicable), and assists with technical issues.
Establish Procedures for Patient Communication	Providers can elicit individual conversations during the session. Peer-to-peer discussion may also occur.	Build in time to discuss challenges and opportunities as a group to create more lines of communication. Develop a process for fielding individual questions.	Promotes a positive group dynamic and a space for patients to get peer and provider feedback/support.
Anticipate Problems with Ongoing Engagement	Providers can elicit individual conversations during the session to address attendance and home program adherence.	Develop a process for patients to synchronously or asynchronously communicate their adherence and any challenges. Develop a process for raising individual questions/concerns. Providers may use a platform that allows them to assign an individualized home program and monitor adherence. Build in time to discuss challenges as a group.	Creative use of technologies to foster group and individual communication with providers, along with built-in group support promotes progress and accountability towards functional goals.

## PROGRAM DEVELOPMENT ADAPTATIONS

### ESTABLISH CRITERIA FOR IDENTIFYING ELIGIBLE PATIENTS FOR GROUP REHABILITATION INTERVENTIONS

Providers can screen for patient eligibility and willingness to participate in group rehabilitation during individual evaluations (in-person or virtually). For in-person groups, eligibility assessment involved determining willingness to participate in group rehabilitation, appropriateness for care delivery/receipt in a group setting, and ability to perform necessary activities with minimal supervision and minimal hands-on assistance. For telerehabilitation groups, eligibility assessments are similar to assessments for in-person groups when considering clinical factors. One necessary difference is the need to evaluate the patient's ability to perform activities with minimal supervision and no hands-on assistance. Non-clinical factors also affect group telerehabilitation eligibility, including technology factors (e.g., equipment availability, internet or cellular capabilities, technology skills) and environmental factors (e.g., home set-up and safety). Finally, we recommend identifying screening criteria that can be reliably administered virtually since the use of telerehabilitation is likely to continue following the COVID-19 pandemic (Hale-Gallardo et al., 2020; Lee, 2020; Prvu Bettger & Resnik, 2020; Quinn et al., 2020). For example, screening tools that require special equipment or large spaces may not be feasible to conduct virtually. Additionally, traditional physical performance tests that use time as the outcome (e.g., 5 times sit to stand) (Guralnik et al., 1994) may not be as reliable as alternative tests that use repetitions or quality metrics as the outcome (e.g., 30-second sit to stand) (Jones et al., 1999) due to bandwidth issues causing delays or interruptions in video transmission (Venkataraman et al., 2020). Considering all these factors, we created a group telerehabilitation eligibility screening battery that may be completed in less than 15 minutes. The general outline with key domains is described in Table 2.

**Table 2:** General Outline of an Eligibility Screen and Needs Assessment for Group Telerehabilitation

Construct	Measure	Criteria for Inclusion	Adaptations to Address
Technology Equipment	Access to an appropriate digital device (Yes/No) Wireless or reliable cellular internet connection (Yes/No) Vitals equipment and monitoring (e.g., blood pressure cuff, pulse oximeter)	Yes to all questions	Provide options to purchase vitals monitoring equipment and resources to seek reimbursement from insurance for purchases; provide education on vitals monitoring.
Physical Space and Privacy	Physical space large enough for rehabilitation interventions (Yes/No) Place to communicate privately (Yes/No)	Yes to all questions	Identify environmental modifications to make available space work (e.g., moving furniture) Suggest alternative locations (e.g., private rooms in a library or community center, friends/family)
Patient Impairment	Visual Impairment (Yes/No) Auditory Impairment (Yes/No) Mobility Impairments (e.g., use of an assistive device) (Yes/No)	No to all: no adaptations needed Yes to one or more: adaptations needed	Visual: patient to use glasses for the session; instruct and provide patient with assistive technology as appropriate (e.g., screen reader); provider wears high contrast clothing and conducts sessions with contrasting clothes against a plain backdrop with good lighting Auditory: patient to apply hearing aids prior to the session; patient can use headphones and/or complete sessions in a quiet room; instruct and provide patient with assistive technology as appropriate (e.g., closed captioning); provider uses a headset or microphone to enhance audio. Mobility: cue for use of an assistive device during the session; provide instructions for care partner to assist.
Cognition	Cognitive test by phone or video (e.g., Montreal Cognitive Assessment-Blind (Pendlebury et al., 2013) or the Telephone Interview for Cognitive Status [TICS] (Brandt et al., 1988))	Case by case basis as the patient may require modifications.	Request that a care partner be present during sessions, if possible. Provide simple cueing and the use of visual aids as needed. Employ compensatory strategies as indicated (e.g., alarms, notes, reminders).
Readiness to Participate	Stages of change algorithm	Contemplation or above	Health coaching referral Motivational interviewing from provider
Technology Capability	Technology skills (e.g. Mobile Device Proficiency Questionnaire [MDPQ](Roque & Boot, 2018)) Access to an email account.	Case by case basis as the patient may require modifications.	Tailor training based on patient needs. Establish email account. Request and train care partner who can assist with technology.

### ESTABLISH AN EMERGENCY PROTOCOL

Providers leading in-person groups can rely on emergency procedures established by their healthcare system or private practice. Since telehealth has been well established in the VHA, emergency protocols and a standardized system to access emergency medical services were in place prior to the pandemic. Four core safety steps are generalizable outside of the VHA: (1) verify the patient's physical location; (2) verify and/or obtain the patient's emergency contact (name and phone number); (3) ascertain if additional individuals are physically present during the session, and; (4) identify the dispatch number for their catchment zone if treating patients who reside outside of the provider's emergency catchment area. For the last item, the dispatch number will most likely be designated as a “non-emergency,” which may increase wait times. In addition to these core steps, we recommend outlining the roles of the lead provider and assistant in the case of an emergency. For example, designate who is responsible for contacting Emergency Medical Services (EMS), who is responsible for staying online with the patient until EMS arrives, and who is responsible for closing the session to the remaining patients and completing their follow-up. Consideration must be taken to ensure patient privacy during and after the emergency.

### IDENTIFY MAXIMUM GROUP SIZE

The optimal size for in-person groups is variable depending on both the intervention(s) delivered and the medical complexity of the patient population. As such, the ratio of patients to providers ranges from 5–10: 1 with a group of 8–10 patients. Similar considerations dictate the size of telerehabilitation groups; however, it is also necessary to consider the telerehabilitation platform capabilities, additional time requirements, and extra safety measures since interventions are not performed in-clinic. As such, we recommend no greater than a maximum 4:1 ratio of patients to providers when delivering active therapy interventions (e.g., exercise and/or balance training). However, if the primary intervention is education-based or slower paced (e.g., Tai Chi, yoga) and/or the patients are low risk (e.g., younger, lack falls risk, less medically complex), a higher ratio of patients to providers could be employed within the constraints of the telerehabilitation platform.

### DELIVER STAFF TRAINING FOR TELEREHABILITATION ADAPTATIONS

Many current providers have extensive clinical training in delivery of in-person care and rely on access to published evidence to identify and interpret rehabilitation tests and measures. However, most providers have little experience with and/or training in virtual delivery of rehabilitation services, particularly in a group format. Developing and delivering standardized training on key elements of the group telerehabilitation program may reduce unwarranted variability in care delivery and give providers the confidence to adapt in-person groups to telerehabilitation. First, an important piece of evaluating the group telerehabilitation program is appropriately screening patients for eligibility and assessing outcomes using tests and measures that can be administered reliably through virtual platforms. Many rehabilitation tests and measures have not been formally validated in a virtual format and, thus in the interim, it is necessary to provide initial and ongoing provider training to standardize the virtual assessment and interpretation of tests and measures. Second, to provide consistent delivery of a group telerehabilitation program, we recommend creating a detailed implementation manual that integrates the practice considerations outlined in this manuscript, details an episode of care (e.g., referral process, scheduling, documentation, billing), and contains the logistics needed to make implementation possible in the context of the specific clinic or healthcare system (contact first author for a copy). Finally, as we outline in the preparation section, an assistant is beneficial to streamlining sessions by providing technical assistance. Thus, we also recommend developing training, an implementation manual, and competency checklists for assistants. Core competencies for assistants may include knowledge of roles and responsibilities, communication plans, and proficiency for technical troubleshooting.

## PROGRAM IMPLEMENTATION ADAPTATIONS

### MEASURE PATIENT RESPONSE TO GROUP TELEREHABILITATION WITH A STANDARDIZED BATTERY OF VIRTUALLY ADMINISTERED TESTS AND MEASURES

As with in-person groups, assessing the success of group telerehabilitation programs requires collection and assessment of patient-level outcomes (e.g., functional status, quality of life, satisfaction with care). We recognize that assessment of any rehabilitation test or measure is more difficult in the virtual setting. For example, some functional tests require equipment or measurement of exact distances that may not be feasible in a virtual format. In addition, tests and measures must be safe for the patient to complete without hands-on assistance. Finally, time-based tests may be inaccurate due to reception lags. Thus, while virtual administration and interpretation of tests and measures may not be ideal, we recommend consistency in the test and measure administration from evaluation to discharge, with careful documentation of procedures so the test is reproducible (i.e., location in home, specific equipment or furniture used).

### CONDUCT PRE-PROGRAM ORIENTATION

Conducting a pre-program orientation for all patients prior to the initiation of group rehabilitation helps lay the foundation for a successful episode of care for the providers and all patients. For in-person groups, this may be conducted as a group and include a tour of and orientation to the physical rehabilitation space and an explanation of patient expectations during group sessions (e.g., respect, privacy). Similar orientations are necessary for group telerehabilitation, yet are likely more efficient if conducted individually given the heterogeneity in patients’ technological understanding and capabilities. We provide all patients a written group telerehabilitation agreement form outlining expectations for conduct that includes details specific to telehealth (e.g., no recording). All patients must verbally agree to abide by the expectations outlined in the agreement to participate in a group format.^1^ In addition to the group agreement, several aspects unique to orientation for group telerehabilitation include: (1) providing education on use of self-monitoring equipment; (2) instructing patients how to use signals during group sessions; (3) troubleshooting any technical issues with videoconferencing platforms (e.g., audio and visual), and; (4) setting-up additional technologies needed during the program, if applicable. Most, if not all, of the pre-program orientation may be performed by an assistant (non-provider). Since communication is more challenging in a virtual format, patients need instruction for signals they can use during the session to indicate they need assistance. For example, patients can repeatedly pat their head or tap their nose to signal they need assistance. The pre-program orientation is also an opportunity to troubleshoot any technical issues prior to initiation of group rehabilitation. The patient can test the virtual platform, verify a stable internet connection, and test the audio and video features. Finally, the pre-program orientation is useful to introduce and set up any additional technologies or applications (apps) that may be used in conjunction with the group session (e.g., home program monitoring and reminders).

### PREPARE FOR THE GROUP TELEREHABILITATION SESSION

Preparation for group telerehabilitation sessions will require more time compared to in-person groups. Telerehabilitation has the following unique elements that must be accounted for and are not applicable to in-person groups: the need for technical equipment, the potential for technical issues prior to and during the session (e.g., audio/visual issues, adjusting camera angle), and the need to verify the safety of patients since they are not located in a controlled environment. As such, providers should avail themselves of their organization's technical support resources such as providing or helping patients navigate the purchase of equipment or software and completing test calls prior to the session. These are largely non-clinical tasks that can be completed by an assistant who can also be present during and after the group telerehabilitation session. For efficiency in patient check-in, we recommend opening the virtual room 15–30 minutes prior to the start of the group session. During this time, the assistant can check-in privately with each patient by calling them. Critical tasks during this check-in are to verify the physical location and emergency contact for the patient in case emergency services need to be contacted. This is also an opportune time to obtain standardized pre-session information (e.g., falls since last session, pain levels), and remind patients about necessary equipment (e.g., therabands, weights) and safety guidelines for the session. To help streamline the introduction to the session and anticipate in-session technical issues (e.g., camera or audio functioning), we recommend providing patients with standardized, written communication (mail or email) in advance of the first group session to outline the directions for accessing the virtual platform, structure of session(s), timing of each section within a session, equipment needs for the session, and basic tips for safety in a virtual format (e.g., self-monitoring, camera positioning, equipment, appropriate dress, safe environment set-up, availability of water, cellphone within reach).

### ESTABLISH PROCEDURES FOR PATIENT COMMUNICATION

Patient communication is necessary for both in-person and telerehabilitation groups as it allows for opportunities to discuss barriers to home programs and solutions to optimize long-term maintenance of self-directed programs. During in-person groups, providers have more opportunities to provide individualized patient education. Additionally, the patients may benefit from peer-to-peer forms of education and discussion about rehabilitation-related challenges, which often occur informally during or immediately after in-person group sessions. To adapt to group telerehabilitation, first, we recommend creating and describing a process for patients to ask impromptu questions throughout the group session (e.g., typing questions in the chat box, emailing the provider after the session). The virtual format may lose informal interactions and, thus, requires intentional effort and upfront expectations on when and how individual questions may be answered. Second, we recommend purposefully scheduling time to deliver patient education and allow for group interaction. We find that an efficient approach to discussions is calling on each participant in turn (i.e., round-robin). Group collaboration can provide support for common challenges, help identify individual solutions, and may help counteract the absence of informal conversations among patients that occur during in-person groups.

### CONDUCTING THE TELEREHABILITATION GROUP SESSION

During in-person group sessions, providers can directly observe and monitor patients while providing hands-on assistance in the controlled clinical environment. Furthermore, patients can often call the provider over for a brief individual consultation or direct questions to their surrounding peers. By comparison, in group telerehabilitation, communication and observation are more challenging given the technology constraints (e.g., small size of video tile), uncontrolled environments, diminished informal interaction, and inability to provide hands-on assistance remotely. We recommend at least two personnel, provider and an assistant (clinical or non-clinical), be present during the session. Outlining the roles and responsibilities of each person prior to implementing a group telerehabilitation program (i.e., in the implementation manual) is essential to smoothly coordinating and communicating between staff during the session. The roles of the assistant are to address any non-clinical questions (e.g., technology) and monitor the chat (if applicable). Sometimes it may be necessary to mute all the patients to reduce excess noise; however, this can create safety concerns if a patient needs assistance. For example, the assistant might notice a designated signal for assist (e.g., tapping the head) and then call the patient on the phone to have a private discussion. This process helps reduce interruptions for the other patients, particularly if the help needed is non-clinical (e.g., audio is not working).

The primary role of the provider is to lead the session through demonstration, instruction, and identification of potential modifications. By demonstrating and modeling rehabilitation interventions throughout the session, providers may maximize patient engagement and foster a positive group dynamic. We recommend simplifying the instructions needed for the interventions by identifying 3–5 simple tasks that can be repeated throughout the session. For example, in physical therapy, we might choose four simple activities that are completed as a circuit for four rounds of one minute per activity with a one minute rest between rounds. We also suggest anticipating and identifying appropriate modifications to make the task easier or harder, so it can be tailored to each individual. For example, if one of the tasks is a step-up, this could be made harder for higher functioning individuals by adding a weight. It could be made easier by changing it to a split-stance squat (e.g., stationary lunge) or a mini-squat; both exercises address the same muscle groups (gluteal and quadriceps) in the same manner (closed chain exercise). Planning for modifications in advance allows providers to quickly help patients identify the task that is best for them and allows for accommodation of a group of patients with varying functional abilities.

### ANTICIPATE PROBLEMS WITH ONGOING PARTICIPATION

In-person and telerehabilitation groups face some similar challenges to patient participation, including attendance, active engagement, and adherence to home programs. However, some challenges are unique to telerehabilitation groups. To help identify patients who are not benefitting from group telerehabilitation, we suggest tracking no-shows or missed visits due to technical issues. Excessive missed visits due to technical issues may indicate the patient could benefit more from in-person rehabilitation sessions. In addition, providers may recommend participants come for in-person sessions if the participant appears to have a change in status or worsening/development of a new condition.

Monitoring home program adherence in the virtual format differs from in-person group rehabilitation as it increases the difficulty of having synchronous one-on-one conversations. This can be potentially addressed by establishing a process for patients to asynchronously communicate their adherence and any challenges they experience. For example, many healthcare systems have secure messaging platforms for patient-provider communication that provide a convenient mechanism to facilitate private conversations outside of the care encounter (Saleem et al., 2020). Additionally, providers may use a platform that allows them to assign an individualized home program and monitor adherence through a provider-facing dashboard. Finally, as discussed above, time can be allotted to discuss challenges and solutions to home program adherence as a group.

## DISCUSSION

We convened a group of expert rehabilitation providers with diverse clinical backgrounds and experiences to identify practice considerations for adapting in-person rehabilitation groups to telerehabilitation groups. This manuscript adds to the literature by providing a guide of practical considerations to immediately put into practice during the pandemic-induced, rapid transition from in-person to telerehabilitation groups. Using the MADI framework (Kirk et al., 2020), we identified adaptations related to program development and program implementation. Salient differences for program development include additional screening criteria to identify appropriate patients for the virtual format, establishing emergency protocols, and providing staff training (providers and assistants). Adaptations for program implementation include developing processes for consistent virtual test administration and scoring, conducting an individual pre-program orientation, spending additional time preparing for the session, developing an implementation manual, and including two personnel during group telerehabilitation sessions. Additional adaptations should include intentionally engaging patients in discussion during built-in group time, and developing processes for patients to virtually communicate to the provider adherence to or challenges with home programs for tailoring.

Further study is warranted to understand the effects of group telerehabilitation on patient-level outcomes (e.g., quality of life, physical function) in different patient populations. Current literature for group telerehabilitation is limited to small feasibility studies and populations with stable conditions (Cox et al., 2018; Hwang et al., 2017; Jennings et al., 2020; Kyrdalen et al., 2014; Lin et al., 2014; Pitt et al., 2019; VanRavenstein & Davis, 2018; Wong et al., 2019). For example, Hwang et al. (2017) demonstrated that a home-based telerehabilitation program for persons with chronic obstructive pulmonary disease (COPD) was not inferior to a center-based, in-person program for functional outcomes (six-minute walk test) and had significantly higher attendance. Tsai et al. (2017) found that compared to usual medical care (i.e., no exercise treatment), patients with COPD in the group telerehabilitation program showed greater improvement in endurance (shuttle test) and self-efficacy. Additional research has been conducted for home-based, virtual group exercise programs (Jennings et al., 2020; Kyrdalen et al., 2014; Wu & Keyes, 2006). However, these populations are significantly different from rehabilitation populations as they generally have good health and stable medical status.

This manuscript has a few limitations. First, restriction of the present work to physical therapy may have limited applicability of these practice considerations and their validation for potential translation across multiple rehabilitation disciplines. At the time the expert team formed in our facility, other rehabilitation disciplines did not have protected, non-clinical time to participate in this project. Second, because we are in the beginning stages of adapting in-person groups to telerehabilitation, we do not yet have insight into the adaptations needed to sustain a group telerehabilitation program long-term.

## CONCLUSION

The COVID-19 pandemic has provided an opportunity to advance research, care delivery, and healthcare policy on the use of group telerehabilitation. Research that responds rapidly to changes in patient and system priorities (Prvu Bettger & Resnik, 2020) is needed to advance the evidence base for group telerehabilitation across different settings and clinical subgroups by determining effectiveness, evaluating implementation, informing scalability, and identifying factors related to sustainability. Our expert team developed practice considerations to adapt in-person rehabilitation groups to telerehabilitation groups to provide rapid guidance during a pandemic and create a foundation for sustainability of group telerehabilitation beyond the pandemic's end.

## Footnotes

1 E-mail the first author for a copy of the agreement to participate in a group format: Allison.Gustavson@va.gov

## APPENDIX

**Patients will be asked to review and orally consent to: Group Telehealth Agreement**

### 1. Confidentiality

I understand the laws that protect the confidentiality of my medical information also apply to telehealth, including group treatment conducted over video telehealth. I understand that the [clinic/system] has instituted procedures and policies to protect my privacy and confidentiality.

I understand that everything said and done in group is confidential. I agree to protect the group confidentiality, by not revealing the names of other members of the group, nor what is said and done in the group. I understand that if I violate this confidentiality, I will be removed from the group.

I understand that there is an exception to this confidentiality that applies to the group provider. The one exception to confidentiality is when the provider believes that I may be a threat to myself or others.

### 2. Risks and Consequences

The [clinic/system] does not record telehealth sessions, including group telehealth sessions, without prior approval. I understand that I will not audio or video record any portion of the treatment session. I acknowledge that while this session will not be audio or video recorded by the [clinic/system], there is a risk that the session *could be* audio or video recorded and disseminated by a group member without knowledge or approval from [clinic/system] or other group members. The consequence for any member audio or video recording any portion of the treatment session will be the removal from the group for violating confidentiality, as well as referral for prosecution to the full extent of federal and local laws. Applicable local laws may include the location of the provider and all members.

### 3. Privacy

Participation in this group is voluntary, and I have the right to withdraw from the group at any time without affecting my right to future care or treatment or risking the loss or withdrawal of any program benefits to which I am otherwise entitled. No group member is ever required to answer any question, to participate in any activity, or to say anything. If I am asked questions or asked to participate in an activity that makes me feel uncomfortable, I understand that I have the right to decline, and I agree not to pressure any other group member to participate if they are uncomfortable. I agree to be in a quiet, private location during my session.

### 4. Dignity

I agree that I will be tolerant, respectful, and supportive of other group members. I will avoid language that stereotypes or is derogatory to others and will provide only helpful feedback. I will be considerate of others who are talking, will give others a chance to talk, and will not engage in side conversations.

### 5. Behavior

Safety is of the utmost importance. Violence or intimidation toward other group members is not tolerated. Gossip and grudges can be very destructive in a group. I agree that if I have something to say to another group member, I will say it to the member directly and in a respectful way rather than talk about him or her with others.

I understand that if the provider believes that I am under the influence of alcohol or other drugs, I will be asked to leave the group.

**I have read the agreement for group sessions and agree to follow it. The provider will note in my medical record that I have received, read and acknowledged this agreement.**

*In developing this consent form it was necessary to use several technical words; please ask for an explanation of any that you do not understand.
